# Fluorescent Carbon Dots for Sensitive and Rapid Monitoring of Intracellular Ferrous Ion

**DOI:** 10.3390/bios12010041

**Published:** 2022-01-14

**Authors:** Le Minh Tu Phan, Thi Xoan Hoang, Sungbo Cho

**Affiliations:** 1 School of Medicine and Pharmacy, The University of Danang, Danang 550000, Vietnam; 2 Department of Electronic Engineering, Gachon University, Seongnam-si 13120, Korea; 3 Department of Life Science, Gachon University, Seongnam-si 13120, Korea; xoanht89@gmail.com; 4 Department of Health Sciences and Technology, GAIHST, Gachon University, Incheon 21999, Korea

**Keywords:** intracellular Fe^2+^ detection, fluorescence turn-off, nitrogen-doped carbon dots, bioimaging

## Abstract

Although iron is an essential constituent for almost all living organisms, iron dyshomeostasis at a cellular level may trigger oxidative stress and neuronal damage. Hence, there are numerous reported carbon dots (CDs) that have been synthesized and applied to determine intracellular iron ions. However, among reported CDs focused to detect Fe^3+^ ions, only a few CDs have been designed to specifically determine Fe^2+^ ions over Fe^3+^ ions for monitoring of intracellular Fe^2+^ ions. We have developed the nitrogen-doped CDs (NCDs) for fluorescence turn-off detection of Fe^2+^ at cellular level. The as-synthesized NCDs exhibit a strong blue fluorescence and low cytotoxicity, acting as fluorescence probes to detect Fe^2+^ as low as 0.702 µM in aqueous solution within 2 min and visualize intracellular Fe^2+^ in the concentration range from 0 to 500 µM within 20 min. The as-prepared NCDs possess some advantages such as high biocompatibility, strong fluorescence properties, selectivity, and rapidity for intracellular Fe^2+^ monitoring, making NCDs an excellent nanoprobe for biosensing of intracellular ferrous ions.

## 1. Introduction

Iron, an essential constituent that is required for the survival of almost all living cells and organisms, is involved in critical biochemical processes such as oxygen transportation, electron transfer reactions in mitochondria, and DNA synthesis and repair [1]. Iron homeostasis is critical to be regulated to prevent iron deficiency or iron overload that may cause reduced oxygen transport and diminished activity of Fe-dependent enzymes or catalyze the formation of highly reactive hydroxyl radicals, oxidative stress, and programmed cell death, respectively [2,3]. Iron dyshomeostasis is implicated in major neurodegenerative disorders such as Alzheimer’s disease [4,5] and Parkinson’s disease [6,7]. The measurement of iron level could play an important role in the early intervention of neurodegenerative disorders, therefore, it is essential to develop simple and rapid method to accurately monitor irons in aqueous solution, living cells and organisms.

Carbon dots (CDs) have emerged as effective nanoparticles that can be an alternative to conventional fluorescent agents for imaging-assisted sensing applications owing to their superior advantages including ease and large-scale production with low cost, excellent photoluminescence with good photostability, easy functionalization, and sufficient biocompatibility [8,9]. To our knowledge, although fluorescent CDs have been widely utilized in the detection of intracellular iron ions based on the fluorescence quenching effects of iron ions [10,11,12,13,14,15,16,17,18,19,20], most of them were developed for the monitoring of trivalent ferric Fe^3+^ ions [10,11,12,13,14,15]. Few reported CDs have been designed for specific detection of divalent ferrous Fe^2+^ ions over ferric ions [18,19,20] and successfully applied to monitor the intracellular Fe^2+^ ions [16,21]. Ferrous ions enhance oxidative stress by catalyzing the production of reactive oxygen species that induce biochemical signaling processes, leading to degenerative diseases such as cancer, Alzheimer’s disease, and Parkinson’s disease [22,23,24]. Therefore, due to the distinctive properties of fluorescent CDs and the necessity of intracellular ferrous ion detection, it is highly desired to successfully explore the Fe^2+^-sensitive CDs for rapid determination of intracellular ferrous ions.

**Figure 1:**
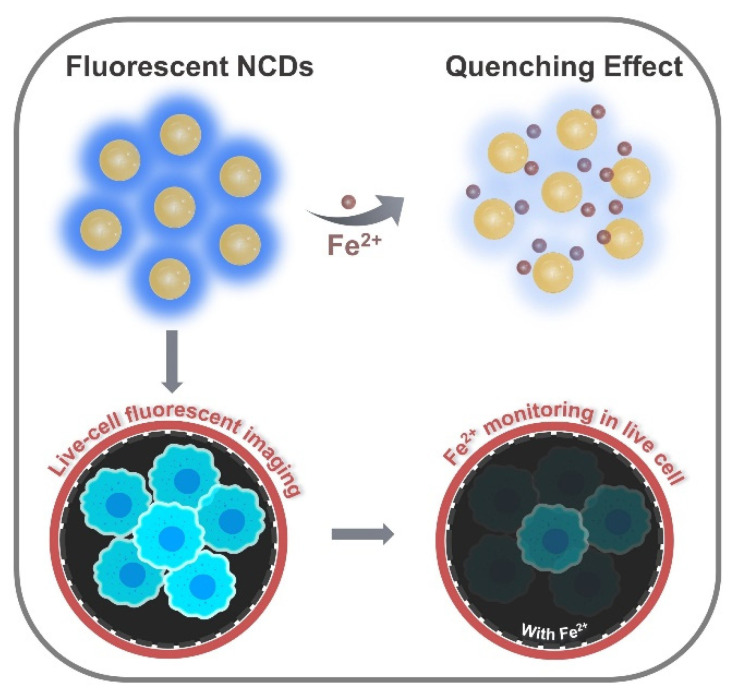
Representation of fluorescence quenching effect of ferrous ions on CDs and their application in intracellular Fe^2+^ ion monitoring.

In this work, we report an affordable nitrogen-doped CDs (NCDs) that could rapidly detect Fe^2+^ ions over Fe^3+^ in aqueous solution and monitor the presence of intracellular Fe^2+^ ions (Figure 1). NCDs have been synthesized by using citric acid as a carbon source and polyethyleneimine molecular weight 1800 (PEI1800) as a nitrogen source. These NCDs exhibit bright blue fluorescence that is responsive towards Fe^2+^ ions and possess low cytotoxicity for bioimaging. The presence of Fe^2+^ causes a strong fluorescence quenching effect on NCDs, due to the coordinate bond formation between Fe^2+^ ions and O atoms on the NCDs surface, showing that NCDs serve as a fluorescent probe for the robust and selective detection of Fe^2+^ in live cells without any modification.

## 2. Materials and Methods

### 2.1. Materials

Citric acid monohydrate (C_6_H_8_O_7_·H_2_O) as carbon source and Polyethylenimine (PEI1800, branched average molecular weight 1800) as nitrogen source were obtained from OCI Company (Seoul, Korea) and Alfa Aesar (Haverhill, MA, USA), respectively. All metal ions were prepared using their nitrate, sulfate, or chloride salts that were purchased from Sigma-Aldrich (Seoul, Korea). Ethylenediaminetetraacetic acid (EDTA) was received from Amresco (Solon, OH, USA). Deionized (DI) water at 18.2 MΩ cm was purified using a Milli-Q system (Purescience, Seongnam, Korea). All reagents were of analytical grade and were used for the experiment without further purification.

### 2.2. Synthesis and Characterization of NCDs

All glassware and autoclave reactors were thoroughly washed with deionized (DI) water and air-dried before being used for the experiment. NCDs were synthesized by hydrothermal treatment of citric acid monohydrate (19 mmol) and PEI1800 (1.25 mmol) in 30 mL DI water. The mixture solution was vigorously stirred for 30 min at room temperature and then heated at 190 °C for 1 h. During short-time hydrothermal synthesis, NCDs with enhanced fluorescence quantum yield were formed with the presence of pyridine-type fluorophore intermediates on the surface [25,26]. After cooling to room temperature, a pale-yellow solution containing NCDs was filtered through 0.22 µm micron filter to remove the large particles. The excess chemicals were removed by using absolute ethanol. Finally, NCDs were dispersed in DI water and stored at 4 °C for further usage.

The as-prepared NCDs were used to characterize the physical properties. High-resolution transmission electron microscopy (TEM-FEI Tecnai, USA) was employed to measure the size of NCDs. Fourier-transform infrared (FT–IR) spectrometry (Jasco 6600FV, Jasco, Tokyo, Japan) was used to explore the surface chemistry of NCDs. Multi-mode microplate reader (96-well microplate, BioTek Synergy H1, Winooski, VT, USA) was applied to acquire the UV–Vis and fluorescent spectra of NCDs. Raman spectrometry (resolution 5 cm^−1^; RamanRxn1, Kaiser Optical Systems, Ann Arbor, MI, USA) with 785 nm laser source (LM-785-PLR-100-1K, Ondax, Monrovia, CA, USA) was used to record the Raman spectra of NCDs before and after addition of Fe^2+^ to explore the mechanism of fluorescence quenching effect of ferrous ions. Fluorescence microscopy (Olympus, CKX53, Tokyo, Japan) was used to observe the fluorescence image of cells with and without incubation of NCDs.

### 2.3. Cell Culture, Cell Cytotoxicity and Cell Uptake

Human keratinocyte HaCaT cell and human breast cancer cell MCF-7 obtained from Korean cell line bank (Seoul, Korea) were cultured in Dulbecco’s Modified Eagle Medium (DMEM) supplemented with 10% heat-inactivated fetal bovine serum and 1% penicillin and streptomycin at 37 °C in 5% CO_2_.

The cytotoxicity of NCDs on HaCaT and MCF-7 cells was determined by EZ-Cytox Enhanced Cell Viability Assay Kit (DOGEN, Seoul, Korea), in accordance with the manufacturer’s instructions. Briefly, 2 × 10^4^ cells/100 µL medium were seeded into 96-well culture plate. Cells were treated with 5 µL of different concentrations of NCDs of (200, 400, 600, 800, and 1000) µg/mL, and incubated for (4 or 24) h at 37 °C. Subsequently, 10 µL of EZ-Cytox solution was added to each well, and cells were incubated at 37 °C for 30 min. The absorbance was measured by a 96-well plate reader at 450 nm.

For cellular uptake assay, 2 × 10^4^ MCF-7 cells or HaCaT cells were seeded into a 96-well culture plate. Cells were treated with 100 µg/mL NCDs for 4 h for cell imaging. The fluorescence images of the cells were obtained by fluorescence microscopy (Olympus, CKX53, Tokyo, Japan).

### 2.4. Turn-Off Fluorescent Probes to Determine Ferrous Ion in Live Cells

The quantitative measurement of ferrous ion concentration correlated with the emission fluorescence intensity of NCDs upon the addition of Fe^2+^ ions. Briefly, 10 µL NCDs solution was diluted in phosphate buffered saline (PBS) buffer to obtain 100 µL solution. Two hundred µL of Fe^2+^ ions at different concentrations of (0–50) µM were added to the as-prepared NCDs solution, and allowed to react for different incubation times of (0–20) min. The fluorescence emission intensity of the resultant solution was recorded to confirm the correlation between Fe^2+^ ions concentration and the change of spectral intensity. The ratiometric analytical process of ferrous ion sensor was validated by calculating the ratio of fluorescence emission intensity before (Fo) and after (F) the addition of Fe^2+^ ions. The interference effect was explored by using the same concentration of other metal ions (Na^+^, K^+^, Ag^+^, Ca^2+^, Pb^2+^, Co^2+^, Mg^2+^, Fe^3+^, Cu^2+^), with EDTA as a masking agent.

The feasibility of NCDs to detect Fe^2+^ in living cells was determined using both HaCaT and MCF-7 cells. Briefly, 2 × 10^4^ cells of HaCaT and MCF-7 cells were seeded into 96-well culture plate. Cells were first treated with NCDs for 4 h for successful penetration of NCDs inside cells. In order to effectively detect Fe^2+^, 50 µL of different concentrations of Fe^2+^ solution (from 0 to 500 µM) was added into each group. After 20 min, the fluorescence images of the cells were obtained by fluorescence microscopy (Olympus, CKX53, Tokyo, Japan), and the correct total cell fluorescence was measured using ImageJ software for the semi-quantitative monitoring of Fe^2+^ in living cells.

## 3. Results and Discussion

### 3.1. Physical Characterization of NCDs

**Figure 2:**
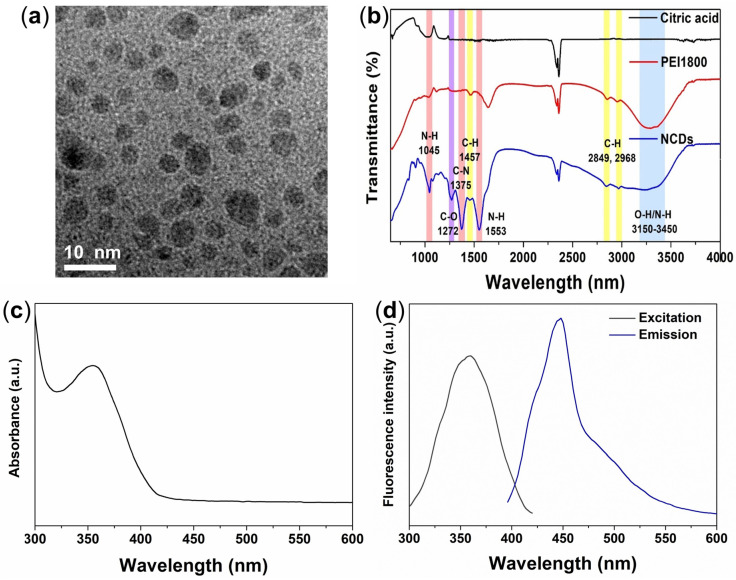
(**a**) TEM image of NCDs. (**b**) FTIR spectra of citric acid, PEI1800 and NCDs for confirmation of surface groups on NCDs. (**c**) UV–Vis absorption spectrum of NCDs. (**d**) The optimal fluorescence spectra of NCDs.

Nitrogen-doped carbon dots were successfully synthesized by a one-step hydrothermal treatment of citric acid and PEI1800 as carbon source and nitrogen source, respectively. The TEM image of NCDs shows the spherical shape of the as-prepared NCDs with an estimated diameter of 3.5–4.5 nm, indicating the high dispersibility and colloidal stability of the NCDs (Figure 2a). The FTIR spectra of citric acid, PEI1800, and NCDs were performed to assess the surface functional groups of the NCDs (Figure 2b). The FTIR spectrum of NCDs showed a broad absorption band at (3150–3450) cm^−1^, which corresponds to stretching vibrations of the –OH and –NH_2_ groups; peak at 1272 cm^−1^, which belongs to the C–O groups; C–H bending vibration peak at 1457 cm^−1^, and C–H stretching regions at (2849 and 2968) cm^−1^; and peaks at (1045, 1553, and 1375) cm^−1^ that are attributed to the N–H and C–N groups that exist in PEI1800. These results thus suggest the presence of hydroxyl, carboxyl, and amino groups on the particle surface that remain from the characteristic groups of citric acid and PEI1800. Optical properties of NCDs were investigated for the further application. Figure 2c shows the absorption peak of NCDs at 365 nm that originated from the n–π transition of C=O bonds [27]. Different excitations were used to monitor the maximum emission of NCDs. Upon optimal excitation at 365 nm, the sharp fluorescence emission peak at 445 nm was obtained, indicating the maximum fluorescence intensity of NCDs at 445 nm under irradiation at 365 nm (Figure 2d). These results indicate the optical and physicochemical properties of NCDs for further sensing and bioimaging application.

### 3.2. Biocompatibility of NCDs as Fluorescent Nanoprobes and Bioimaging Application

**Figure 3:**
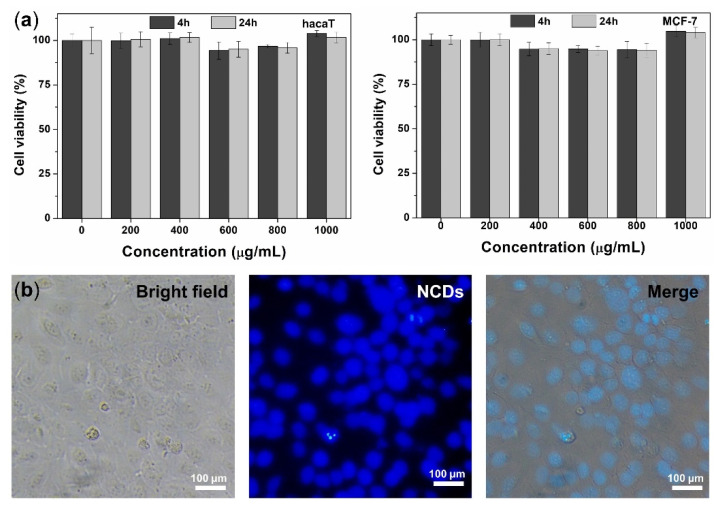
(**a**) Cytotoxicity of NCDs at different concentrations (0–1 mg/mL) toward HaCaT and MCF-7 cells. (**b**) Bright field and fluorescence microcopy images of NCDs treated on MCF-7 cells after incubation at 37 °C for 30 min.

To validate the biocompatibility of the NCDs, the cytotoxicity study was carried out using Ez-Cytox assay on two different cell lines, HaCaT and MCF-7. We chose two reaction time points of (4 and 24) h to observe the cell viability under the treatment of a wide range concentration of NCDs of (0, 200, 400, 600, 800, and 1000) µg/mL. Figure 3a shows that after either (4 or 24) h incubation, the proliferation of both HaCaT and MCF-7 was almost unaffected by NCDs, even at the concentration of NCDs up to 1000 µg/mL. The result reveals that the NCD probe is highly biocompatible, and could be applied for intracellular imaging. Owing to their low cytotoxicity, the ability of the NCDs as optical nanoprobes to operate in in vitro living cell imaging was also evaluated. The applicability of NCDs for intracellular fluorescence imaging was investigated in live MCF-7 cells. It was found that the cells incubated with NCDs exhibited strong blue fluorescence (Figure 3b), demonstrating that the NCDs could penetrate the cell membrane, and translocate into cells. This result indicates that the NCDs are potential optical nanoprobes for real-time cell imaging and tracking.

### 3.3. Rapid Detection of Fe^2+^ Ion

**Figure 4:**
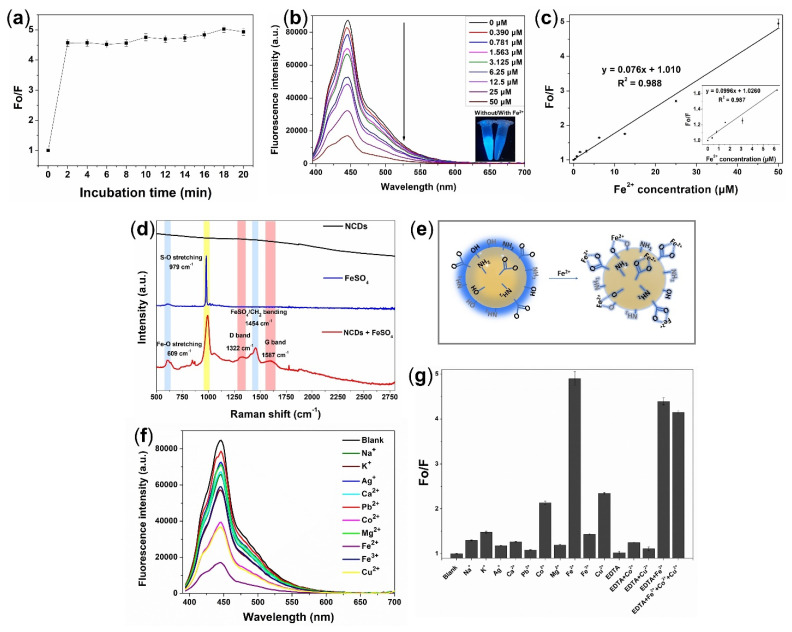
(**a**) Fluorescence quenching efficiency of NCDs in the presence of Fe^2+^ at different incubation time. (**b**) Fluorescence quenching spectra under 365 nm excitation in the presence of increasing concentrations of Fe^2+^ ion of (0–50) µM. Inset: photographs of NCDs solution with/without ferrous ion under UV light at 365 nm presents the quenching effect of Fe^2+^ ion. (**c**) Linear correlation between Fe^2+^ concentration of (0–50) µM and fluorescence quenching efficiency (Fo/F). Inset: linear relationship between Fe^2+^ concentration of (0–6.25) µM and quenching efficiency with good linear calibration. (**d**) Raman spectra of NCDs, FeSO_4_, and NCDs with Fe^2+^ ion reveal the charge–transfer fluorescence quenching by chelation between NCDs and Fe^2+^. (**e**) Schematic of chelating quenched fluorescence via Fe^2+^ chelation and electron transfer. (**f**) Fluorescence spectra of NCDs after addition of various metal ions. (**g**) Quenching effect of different ions on fluorescence of NCDs.

To quantitatively evaluate the detection ability of NCDs to Fe^2+^, the fluorescence intensity changes of an equal amount of NCDs in the presence of different concentrations of Fe^2+^ ion were measured. In order to determine the optimal quenching time, the fluorescence intensities of NCDs were evaluated at different times after addition of Fe^2+^ ion. The value of fluorescence intensity was obtained from the fluorescence spectra by recording the maximum fluorescence emission intensity at 445 nm under 365 nm excitation. Figure 4a shows that during the early stage of reaction (just 2 min after the quenching reaction), the fluorescence quenching ratio Fo/F (where Fo is the fluorescence of the initial NCDs, and F is the fluorescence after the addition of Fe^2+^) was dramatically increased (approximately 4.5-fold increase); and after 20 min, this quenching effect remained constant. Thus, 2 min was chosen for the following experiments. Under the optimal live-cell culture conditions (pH 7.4), there is a continuous decrement in the fluorescence signals of NCDs when the concentration of Fe^2+^ increases from (0 to 50) µM. In the presence of 50 µM Fe^2+^, the fluorescence emission of the as-prepared NCDs is almost completely quenched (Figure 4b). These results imply that the fluorescence of NCDs is highly sensitive to the Fe^2+^ concentrations in the solution. The Fo/F value increases with the concentration of Fe^2+^ ion, and displays a good linear relationship in the concentration range (0–50) µM with a correlation coefficient (R^2^) of 0.988. The low limit of detection at 0.702 µM was calculated by 3S/b where S and b are the standard error value of intercept and the slope value of the calibration curve, respectively (Figure 4c).

The possible quenching mechanism of Fe^2+^ was confirmed by Raman spectra. The Raman experiment was performed to provide more insight into the chemical nature of the as-prepared NCDs before and after interaction with Fe^2+^. Figure 4d shows that in the absence of Fe^2+^, NCDs exhibit no Raman signal under the measurement condition, due to the strong background fluorescence emission [28]. The presence of Fe^2+^ provokes a change of the NCDs’ surface chemistry via chelation, due to the coordinate bond formation between Fe^2+^ ions and O atoms on the surface functional groups of NCDs [29]. The Raman spectrum of FeSO_4_ clearly shows a sharp peak at 979 cm^−1^ attributed to the presence of S–O stretching bond [30,31,32]. The Raman spectrum of the NCDs after the treatment of Fe^2+^ ions indicated that the peaks at (1322, 1587, and 1454) cm^−1^, known as the disorder (D) band, crystalline (G) band [33,34], and -CH_2_ stretching vibration [35], respectively, are more prominent compared to the non-treated NCDs, suggesting the initial characteristics of the NCDs appear after the removal of the fluorescence background due to the quenching fluorescent effect of Fe^2+^ ions. In addition, a prominent peak found at 609 cm^−1^ in the Fe^2+^-treated NCDs is attributed to the formation of the Fe–O coordinate bond due to the coordinate bond formation between the Fe^2+^ and O atoms on the surface of NCDs, while a weak peak in the same region was seen for FeSO_4_ alone. These results confirmed the fluorescence quenching mechanism of Fe^2+^ toward NCDs due to the chelation of NCDs with Fe^2+^ that is attributed to the interaction between abundant functional groups of NCDs and Fe^2+^, where the electron transfer to Fe^2+^ ions occurred, in which NCDs serve as a donor of electron pairs, while the Fe^2+^ is the acceptor, leading to fluorescence quenching, as illustrated in Figure 4e.

To support the selectivity of the NCDs nanoprobe in detecting Fe^2+^, the fluorescence response of NCDs toward Fe^2+^ over interferent ions has been investigated. The same concentration of NCDs was treated with Fe^2+^ and other metal ions of Fe^3+^, Na^+^, K^+^, Ag^+^, Ca^2+^, Pb^2+^, Co^2+^, Mg^2+^, and Cu^2+^. Figure 4f,g show that among all these metal ions, the NCDs treated with Fe^2+^ show a remarkably high Fo/F value, implying the strong quenching effect on NCDs fluorescent intensity, while less fluorescence quenching effect was observed in NCDs treated with other metal ions. Especially, a negligible quenching was obtained with Fe^3+^ ion, indicating the high selectivity of these NCDs to Fe^2+^ over Fe^3+^. Co^2+^ and Cu^2+^ ions caused a slight quenching interference effect on NCDs; however, upon the addition of EDTA as a masking agent, these ions were effectively masked. Due to rapid reaction, the quenching effect caused by Fe^2+^ was not affected by the presence of EDTA. These results demonstrate that the NCDs fluorescence probe is selective toward Fe^2+^ over the other metal ions due to the complex formation between Fe^2+^ and NCDs. The presence of other metal ions does not significantly affect the quenching effect of NCDs toward Fe^2+^, illustrating the selectivity of NCDs towards Fe^2+^ in a complex ions sample.

### 3.4. Turn-Off Fluorescent Probe for Fe^2+^ Monitoring in Live Cells

**Figure 5:**
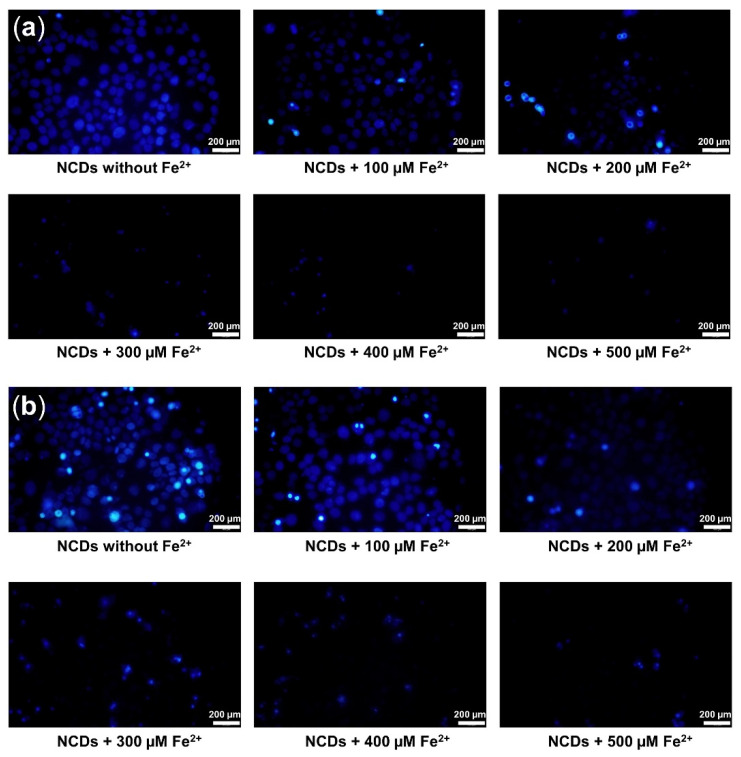
Fluorescent images of NCDs pre-incubated MCF-7 cells (**a**) and HaCaT cells (**b**) after the introduction of different concentrations of Fe^2+^ ion for 20 min under 365 nm excitation.

**Figure 6:**
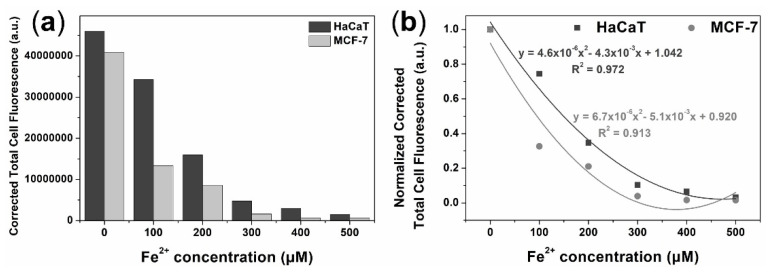
(**a**) Semi-quantification of fluorescence staining intensity for the monitoring of intracellular ferrous ion in HaCaT cells and MCF-7 cells. (**b**) Correlation between intracellular Fe^2+^ ion concentration and normalized corrected total cell fluorescence.

The feasibility of the proposed NCD probes in live cells was evaluated by monitoring the level of intracellular Fe^2+^ by fluorescence microscopy using HaCaT and MCF-7 cell lines. Upon the addition of Fe^2+^ at (0–500) µM concentrations, the fluorescence intensity of the NCDs solution was gradually quenched in both MCF-7 cells and HaCaT cells (Figure 5). Quantitative analysis of the fluorescence intensity in Fe^2+^ treated MCF-7 and HaCaT cells showed a significant decrease of the fluorescence signal in accordance with the increase of Fe^2+^ concentrations (Figure 6a). A significant correlation between the increased concentration of intracellular Fe^2+^ and the reduced cellular fluorescence was found in both MCF-7 and HaCaT cell lines, with the coefficient value (R^2^) of (0.972 and 0.913), respectively (Figure 6b). The fluorescence quenching ability of Fe^2+^ was found to be more efficient in the breast cancer cell MCF-7, compared to keratinocyte HaCaT. Due to the high demand of iron, the transferrin receptor is overexpressed at the surface of cancer cells to facilitate iron influx into cancer cells, leading to more efficient quenching effect on the fluorescence of NCDs, as compared to normal cells [36,37]. As the results, the capacity of NCDs to detect Fe^2+^ in different cells via fluorescence quenching could exhibit their potential in monitoring of intracellular Fe^2+^.

## 4. Conclusions

Fe^2+^-sensitive CDs have been successfully designed to determine the presence of intracellular Fe^2+^ ions based on quenching effect upon addition of Fe^2+^ into CDs. The NCDs not only possessed the strong blue fluorescence with high biocompatibility for bioimaging application, but exhibited the potential ability to rapidly detect Fe^2+^ ions over Fe^3+^ ions in aqueous solution within 2 min at low limit of detection of 0.702 µM. Additionally, these NCDs were successfully applied as an effective probe for the monitoring of Fe^2+^ in living cells including normal and cancer cells within 20 min. The fluorescence turn-off detection of Fe^2+^ at cellular level was easily observed within short time, suggesting the feasibility of these CDs for their utilization as potential probes to monitor intracellular Fe^2+^. There is less report about Fe^2+^-sensitive CDs for intracellular sensing of Fe^2+^, thus, these CDs could significantly contribute to further intracellular Fe^2+^ sensing application by acting as promising candidate for bioimaging and rapid monitoring of intracellular Fe^2+^ owing to their advantages including ease of synthesis, strong fluorescence properties, high biocompatibility, and selective and rapid sensing performance of Fe^2+^ in living cells.

## Data Availability

The dataset generated and analyzed in this study is not publicly available, but may be obtained from the corresponding author upon reasonable request.
